# Supplementary figures and images for: Saliva molecular testing bypassing RNA extraction is suitable for monitoring and diagnosing SARS-CoV-2 infection in children
Source: PLoS One. 2022 Jun 15;17(6):e0268388. doi: 10.1371/journal.pone.0268388 (PMC9200166; doi:10.1371/journal.pone.0268388)

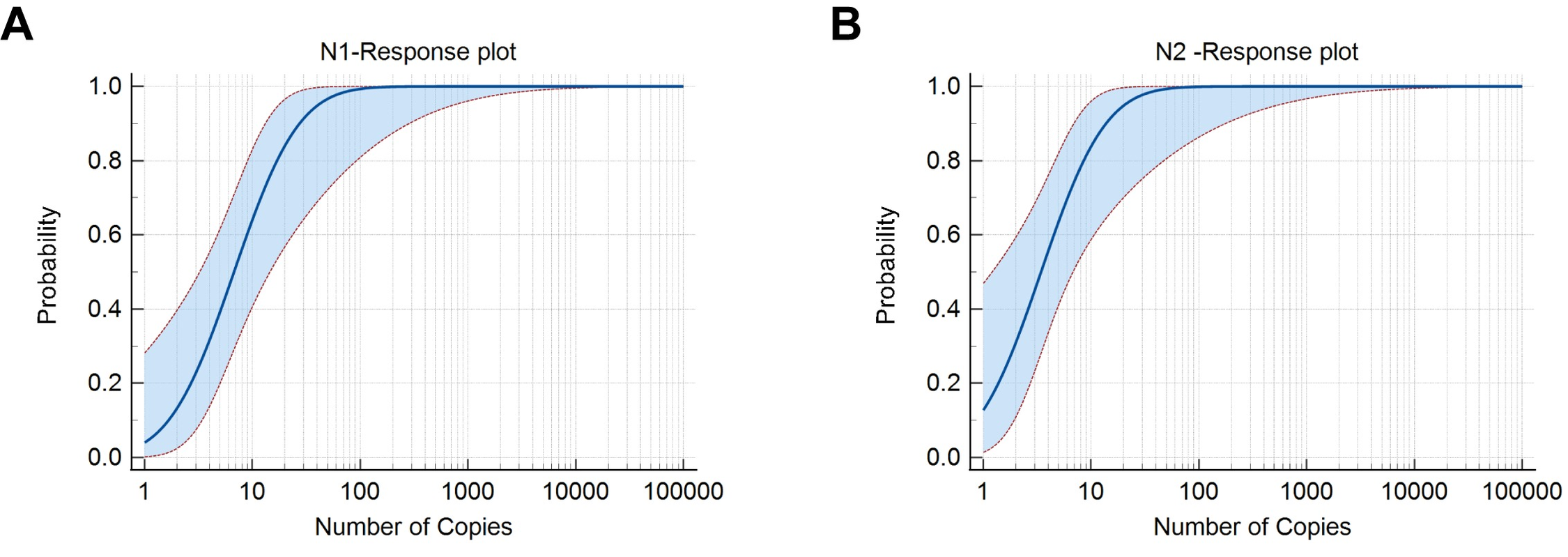

Supplement: S1 Fig — Probit analysis (plot of fitted model) was performed using the MedCalc software to determine the LoD by fitting template copies (on a logarithmic scale) against the cumulative fractions of positive PCR observations (blue line), and to calculate the lower and upper 95% confidence intervals (CI) (red dashed line). LoD for N1 probe was defined at 40.74 copies with a 95% CI of 17.54–738.16 (A), and at 20.39 copies for N2 probe with a 95% CI of 9.21–547.17 (B). Detection linearity and correlation between CTs and copy number was observed between 20 and 20000 copies (data not shown). (TIF) [file pone.0268388.s001.tif]

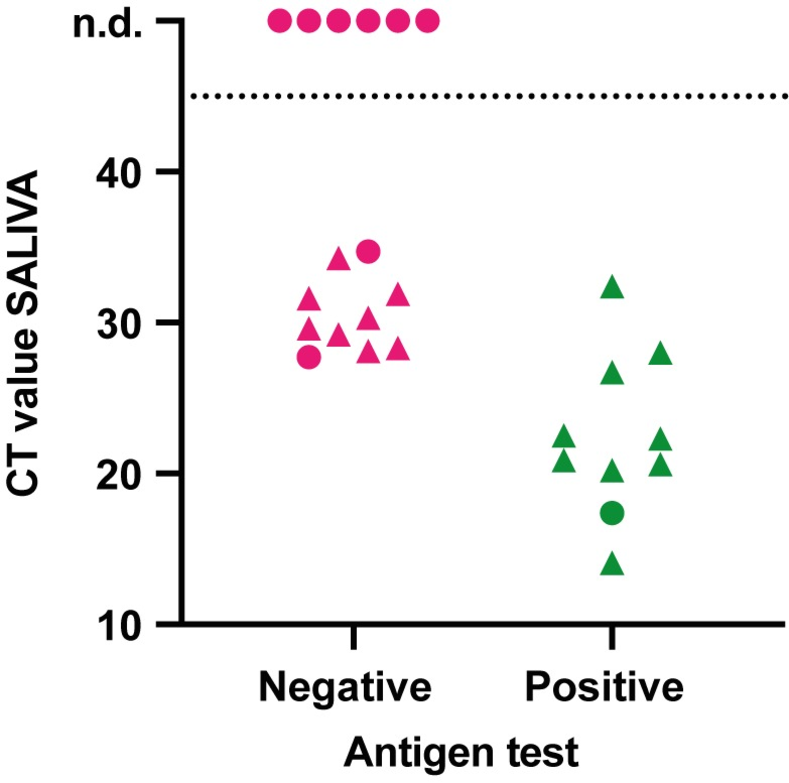

Supplement: S2 Fig — Graphical representation of CT values in saliva after RNA extraction vs. rapid antigen test result, with dots representing symptomatic children, triangles asymptomatic patients. n.d., not-detected. The COVID-19 antigen rapid test from ALL TEST (ref. ICOV-502) was used accordingly to the manufacturer’s instructions, with the exception that instead of NP swab, saliva samples diluted 1:2 in extraction buffer were used. (TIF) [file pone.0268388.s002.tif]
